# The Welfare of Cows in Indian Shelters

**DOI:** 10.3390/ani9040172

**Published:** 2019-04-16

**Authors:** Arvind Sharma, Uttara Kennedy, Catherine Schuetze, Clive J. C. Phillips

**Affiliations:** 1 Centre for Animal Welfare and Ethics, School of Veterinary Science, University of Queensland, Gatton, QLD 4343, Australia; c.phillips@uq.edu.au; 2 RSPCA Queensland, Wacol, QLD 4076, Australia; uttaram@yahoo.com; 3 Faculty of Arts and Social Science, The University of Sydney, Sydney, NSW 4343, Australia; vajracat@gmail.com

**Keywords:** India, cow shelters, gaushala, welfare, assessment

## Abstract

**Simple Summary:**

The welfare of cows in traditional cow shelters (gaushalas), was assessed on the basis of the measurement of animal- and resource-based welfare parameters and description of the herd characteristics by the manager. A description of the condition of the cows and the resources provided to them is provided in this cross-sectional study. Small space allowance per cow, non-uniform flooring, little freedom of movement, and lack of access to pastures were the key welfare issues observed in the study. Very few cows were recorded as lame, but about half had carpal joint lesions and slightly less had lesions from interacting with shelter furniture. This study will inform the stakeholders about the concept of welfare auditing of the cow shelters, for better welfare and management of the cows in the shelters.

**Abstract:**

Cow shelters (gaushalas) are unique traditional institutions in India, where aged, infertile, diseased, rescued, and abandoned cows are sheltered for the rest of their life, until they die of natural causes. These institutions owe their existence to the reverence for the cow as a holy mother goddess for Hindus, the majority religion in India. There is a religious and legal prohibition on cow slaughter in most Indian states. A cross-sectional study was conducted to assess the welfare of cows in these shelters, which included the development of a welfare assessment protocol, based on direct animal-based measurements, indirect resource-based assessments, and description of the herd characteristics by the manager. A total of 54 cow shelters in 6 states of India were studied and 1620 animals were clinically examined, based on 37 health, welfare, and behavior parameters. Thirty resources provided to the animals, including housing, flooring, feeding, watering, ease of movement, cleanliness of facilities, lighting, temperature, humidity, and noise levels in the sheds were measured. The study showed that the shelters contained mostly non-lactating cows, with a mean age of 11 years. The primary welfare problems appeared to be different to those in Western countries, as the major issues found in the shelters were facility-related—the low space allowance per cow, poor quality of the floors, little freedom of movement, and a lack of pasture grazing. Very few cows were recorded as lame, but about one half had carpal joint hair loss and swelling, and slightly less had lesions from interacting with shelter furniture. Some shelters also had compromised biosecurity and risks of zoonosis. These issues need to be addressed to aid in ensuring the acceptability of these institutions to the public. This welfare assessment protocol aims to address the welfare issues and problems in the shelters, by providing feedback for improvement to the stakeholders.

## 1. Introduction

India has the largest cattle population in the world, with more than 190 million cattle [1], used primarily for dairy and draft purposes. Most rural people own a few cows but have limited land for grazing, especially as the human population has encroached upon their traditional grazing lands, leading to cows roaming freely in the streets and causing traffic problems. In some states, crop raiding by street cattle has led to significant human-animal conflict [2], and there are many fatal road accidents involving cattle on the streets [3,4,5].

The majority of the Indian population follow Hinduism, which has strong influences on animal husbandry, in particular, on euthanasia. Euthanasia of species of animals, other than cattle, is considered and carried out by registered veterinarians. However, whilst euthanasia in cows in extreme cases is allowed under the law and is condoned by the Animal Welfare Board of India, it is culturally problematic and, therefore, not often practiced [6,7,8]. Street cattle overpopulation is an emerging social and public health problem, especially, in the light of the prohibition of cow confiscation and slaughter in most states [9]. The large cattle population of India is also partly, due to the ancient tradition of sheltering, feeding, and caring for cattle, after they have ceased production [10]. In most Indian states there are cow shelters or sanctuaries, termed ‘gaushalas’, or for more recent shelters ‘go sadans’ (hereafter, collectively termed ‘shelters’), where abandoned, infertile, and chronically ill cows are sheltered by philanthropists, animal protection organizations, religious organizations, and religious temple trusts. 

Shelters play a significant role in the management of stray cattle in India [11], but might have inadequate space, leading to unhygienic conditions [12,13]. Transfer of cattle between shelters is rare, usually only occurring if a single organization manages several shelters. There are, approximately, 3000 care shelters for old and infirm cows [14], though the exact number is not known [11]. There are 1837 gaushalas funded by the Government of India, through a central statutory body—the Animal Welfare Board of India (AWBI) [15]. The AWBI provides funds for the management and infrastructural needs of cows in affiliated shelters. 

No scientific assessment of the welfare of stray and abandoned cows in shelters has yet been attempted, apart from the testing of vaccines against paratuberculosis [16,17]. As a result, there are no audits, although protocols for the assessment of the welfare of dairy cattle have been developed and validated, using a field-based protocol that is mainly relevant to Western production systems [18,19,20]. There is a lacuna in the literature and in the Indian animal industries, generally, about the use of indicators to assess welfare in non-productive shelter cows. It is sometimes assumed that the welfare of the cows in gaushalas is worse than those kept in farms under semi-intensive or intensive conditions, as the cows have outlived their commercial utility [21], and they just bear a sentimental value for the Indian society. In this study, we had the objective of measuring relevant aspects of welfare, using an assessment protocol similar to that used for commercial cattle enterprises. 

## 2. Materials and Methods 

We first reviewed the animal, resources, and the management-based measures used to assess cattle welfare in different welfare assessment protocols for dairy and beef cattle industries. Potential measures were discussed with a group of experts selected by us (animal welfare scientists (*n* = 4), veterinarians (*n* = 4), veterinary epidemiologists (*n* = 2 and veterinary clinicians (*n* = 2)) in a one-day stakeholder workshop in Delhi in November 2016. Each identified measure was considered for its relevance to a typical Indian sheltered cow scenario. The Welfare Quality^®^ Protocol [22] objective of good feeding, housing, health, and appropriate behavior, was used as the guiding directive. As a result of the discussions, 37 animal-based, 31 resource-based, and 35 management-based measures were selected, which were considered relevant, feasible and suitable for an on-field welfare assessment of cow shelters. Most of the animal-based measures selected for this assessment had been tested and validated in previous welfare assessment studies on cows [18,19,20,22,23,24,25,26]. 

The study was endorsed by the AWBI, which provided the contact details of 34 shelters. The animal assessment component was approved by the Animal Ethics Committee of the University of Queensland (Approval Number: SVS/CAWE/314/16/INDIA). The assessments took place from December 2016 to July 2017. A power analysis [27] indicated that a sample size of 50 shelters would adequately represent the shelters in the major Indian states. Hence a total of 54 cow shelters were selected from 6 states of India, five of which have the predominant cow shelter population in India (Gujarat, Maharashtra, Rajasthan, Punjab, and Haryana) and one state (Himachal Pradesh), which was at that time establishing many new cow shelters [28]. Following discussion with key stakeholders, the criteria for shelter inclusion in the study were—a minimum of 30 cows, that it was not a commercial dairy unit (where commercial indicates that more than 20 L milk per day was being sold), and that the shelter was managed by a philanthropic, temple, government, or public trust. Out of the 54 shelters, 26 shelters were visited on the advice of state veterinary officers, which fell within their administrative jurisdiction and the AWBI, and the remaining shelters were obtained using a snowballing technique, taking recommendations from shelter managers that were visited by the two field researchers in this study (AS and UK). There was no significant difference (*p* < 0.05) between shelters obtained by the two methods in any measured parameter, when compared by analysis of variance or a Moods median test (in the case of non-normal residuals in the ANOVA model). 

Within each animal shelter, resource and manager-based assessments were conducted. For the animal-based assessment, 30 animals were selected per shelter, as recommended, following a power analysis. Only primiparous and multiparous cows were selected; calves, bulls, steers, or preparturient heifers were not selected. This selection of cows was the same for each shed, within a shelter—every third cow in a line, group, or side of a shed, was selected, irrespective of the distance between them, up to a total of 30. In the case of the different lines of tethered cows or cows being housed in more than one group, an equal number of cows was selected from each line, group, side of a shed (where it was bisected by a passage) or shed (if >1). The assessments for the animal-based measures took place on one day in each gaushala, beginning at 09:00 hours, approximately one hour after the cows were fed.

Pilot trials were also done to validate the chosen measures in the two shelters before the commencement of the actual data collection. One of the researchers (AS), a veterinarian, led all the assessments in the shelter. A second veterinarian (UK) assisted with the resource-based measurements in the 12 shelters. If there was more than one shed in a shelter, cows in a maximum of the two sheds were measured.

### 2.1. Interview with the Shelter Manager

The shelter visit started with an interview with the shelter manager, using prepared questions. These included the total number of cattle in the shelter, the types of cattle shed, annual mortality rate, provision of pastures for the cows (dichotomous, present, or absent), mean daily time (hours per day) spent by cows at pasture and yards, and source of water supply (municipal, well, natural, or potable water supply). Shed cleaning method and schedule [29,30], feeding schedule, fodder type, variety and quantity fed to the cows, were both recorded from the interview, and confirmed by visual inspection of the premises. The shelter manager was asked what the vaccination schedule was for cows in the shelters; whether raw milk or urine was sold (the former to confirm the selection of the shelter according to our criterion); and about the deworming protocol, disposal of dung, use of veterinarians’ services, disposal of carcasses, biosecurity measures, and disease outbreaks over the last five years.

### 2.2. Animal-Based Measures

One of the authors (AS) underwent a two day low-stress livestock handling course and a three month training, in scoring the cows for assessment of body condition, lameness, claw overgrowth avoidance distance, dirtiness, limb lesions (joint hair loss, ulceration and swellings), skin lesions, rumen fill, fecal consistency, and rising behavior, at the School of Veterinary Science, The University of Queensland. The age, breed (classified as indigenous, crossbred with indigenous breeds, crossbred with exotic breeds, such as Holstein Friesian or Jersey, or exotic), lactation status, presence or absence of horns and presence or absence of identification (ear tags, branding marks) were ascertained from a general inspection of each animal, an oral examination, and discussion with the manager. In our study each cow was restrained for the animal-based measurements, restricting the expression of temperament. Therefore, each sampled cow’s temperament was assessed during restraint on a simple dichotomized scale (docile or aggressive), which was loosely derived from a five-point scale [31], for loosely restrained cattle in a particular area of the barn. The Cow Comfort Index (CCI) [32], was modified for shelter cows, by counting the number of cows lying down in the sheds, described as a proportion of the total in the shed. The animal-based measures used in the study have been summarized in Appendix A Table A1.

### 2.3. Measures on Selected Cows

The avoidance distance (AD) was assessed at the beginning of each shelter visit, one hour after morning feeding, as prescribed by the Welfare Quality^®^ protocol [33]. A cow was approached from immediately in front of each animal, at a rate of 1 step per second, starting at 2 m from the manger. The distance between the assessor’s hand and the cow’s head was estimated at the moment the cow moved away or turned its head, in the following four categories—touched, and hand within 50, 51–100 cm, and >100 cm. For each shelter, the median AD classification and percentage of cows which could be touched on the head were calculated. In the shelters where cows were tethered, they were untied and moved outside the shelter, to assess AD and lameness, and then retied for all remaining animal-based measures. Body Condition Score (BCS) was determined using a 1–5 scale [34,35], and scored to quarter points. A cow with a score of ≤ 1.25 was considered emaciated, 1.5–2 was labeled ‘thin’, 2.25–3.75 was labeled ‘normal’, and 4 or more was labeled ‘obese’.

Lameness scores were attributed using a numerical rating scale for walking cows [36]: ‘1’—‘not lame’ (smooth and fluid movement); ‘2’—‘mildly lame but not easily observable’ (an imperfect gait but able to freely move with a mildly arched back); ‘3’—‘moderately lame’ (able to move but not freely, with an arched back); ‘4’—‘lame’, (unable to move freely with an asymmetrical gait and abnormal head movement); ‘5’—‘severely lame’ (severely restricted in movement, requiring considerable encouragement to move, and a severely arched back). Claw overgrowth was assessed by the visual inspection of each sampled cow, using a four-point scale [37]: ‘0’—‘normal claws’; ‘1’–‘3’—representing ‘mild’, ‘moderate’, and ‘severe’ claw overgrowth, respectively.

Rising behavior of a sample of 30 cows that were lying down in each shelter was categorized using an existing protocol [38,39]. All cows lying in the shelter were coaxed to get up with the use of a minimum amount of force. If the presence of the assessor did not evoke rising (as happened with four cows), they were given one or two moderate slaps on the back, followed by more forceful ones if necessary. Rising behavior was categorized as follows: ‘1’—‘normal’ (smooth and a normal sequence of rising behavior); ‘2’—‘easy, but slightly interrupted’ (smooth movement with slight twisting of the head but with normal sequence of rising process); ‘3’—‘uneasy, with effort’ (sudden movement and difficulty in rising with awkward twisting of the head and neck, but following a normal sequential rising process); ‘4’—‘abnormal’ (uncharacteristic sequence of a rising event); ‘5’—‘refused to get up’. Rising restrictions caused by the shelter facilities were scored according to a four-point scale [40]: ‘0’—‘unrestricted’ (cow is able to rise as if it were in a pasture); ‘1’—‘mild restrictions’ (cow is able to modify standing to rise comfortably as it lunges sideways and not forwards); ‘2’—‘cow takes time to rise and hits shed fixtures or fittings while rising’.

Swellings, hair loss, and ulcerations on the hock and carpal joints were scored according to an established scale [41,42]: ‘1’–‘3’, representing ‘mild’, ‘medium’, and ‘severely’ swollen joints, respectively. Hock joint hair loss and ulceration were described on a similar scale [41,42]: ‘0’—‘no hair loss or ulceration’; ‘1’—‘mild hair loss or ulceration < 2 cm^2^’; ‘2’—‘medium hair loss or ulceration, approximately 2.5 cm^2^’; ‘3’—‘severe hair loss or ulceration > 2.5 cm^2^’. Carpal joint injuries were scored as: ‘0’—‘no skin change’; ‘1’—‘hairless’; ‘2’—‘swollen’; ‘3’—‘wound’ [41]. 

Dirtiness of the hind limbs, udder, and flanks was classified by visual inspection of the cows from the left, right side, and from behind [43]: ‘1’—‘no dirtiness’; ‘2’—‘mildly dirty’ (small soiled areas of dirtiness with no thick scabs); ‘3’—‘medium dirtiness’ (large soiled areas but with < 1 cm thick scabs of dung), and ‘4’—‘severely dirty’ (large soiled areas with > 1 cm thick dung scabs). The condition of the coats of the sampled cows was assessed on a slightly modified (from the reference scale) 3-point scale [44] as: ‘1’—‘dull and short’; ‘2’—‘shiny and short’; ‘3’—‘dull and hairy’. Ectoparasitism was assessed through a modification of the scoring pattern devised by Popescu et al. [45]: ‘1’—‘absence of ectoparasites’; ‘2’—‘mild infestation’ (no lesions, not easily visible by the naked eye, only on tactile perception in the neck region); ‘3’—‘moderate infestation’ (visually observable ectoparasites or immature forms or eggs in the neck, groin, perirectal, tail root and switch regions); ‘4’—‘severe infestation’ (observable mature ectoparasites over much of the body, especially regions mentioned in score 3).

Lesions were predominantly acquired from shelter furniture as a consequence of interaction with sharp nails/metals protruding from shelter gates, broken mangers, broken edges of shed walls, barbed wire fencing, and manifested in the form of hair and tissue loss. Sharp lacerations and avulsion of the skin were described by using a 3-point scale [37]: ‘0’—‘normal’ (no lesions present); ‘1’—‘small area of hair loss’; ‘2’—‘moderate area of hair loss or thickening of the skin’; ‘3’—‘severe’ (a large area of hair loss or breakage of the skin). Other skin lesions or integument alterations were recorded as: ‘0’—‘normal’ (no apparent lesions); ‘1’—‘mild hair loss’ (<2 cm^2^); ‘2’—‘moderate’ (>2 cm^2^ hair loss and inflamed skin); ‘3’—‘severe’ (a large >4 cm^2^ area of hair loss with extensive skin inflammation and breakage) [46].

The protocols for teat and udder scoring, skin tenting time, and presence of oral lesions, were designed by the authors, because it was anticipated that emaciation, teat, and udder abnormalities, oral infections, and the presence of very old cows would be more common in the shelters than in dairy cow farms, for which other scales have been developed. The assessment of skin turgor in cattle is a measurement of the time a skin tent takes to return to its original position and is a practical way of assessing dehydration [47]. It was assessed with the following scale: ‘1’—‘≤2 s’; ‘2’—‘>2 s ≤ 6 s’; ‘3’—‘>6 s’. The scales for other parameters were, oral lesions: ‘0’—‘absent’, ‘1’—‘present’; teat and udder: ‘1’—‘normal teats and udder’; ‘2’—‘dry udder and teats’, ‘3’—‘teat cracks’, ‘4’—‘warts on teats and udder’; ‘5’—‘acute lesions on the teats and udder’; ‘6’—‘chronic lesions on teats and udder’. 

Neck lesions were classified as: ‘1’—‘no observable skin change’; ‘2’—‘hair loss’; ‘3’—‘swollen’; ‘4’—‘closed wounds’ (hematomas or closed abscesses); ‘5’—‘open wounds’ [48]. Respiratory problems were measured as the presence or absence of coughing in any of the 30 cows sampled in the sheds, during the total examination period of the sampled cows in each shed. Ocular lesions, nasal discharge, hampered respiration, diarrhea, and vulvar discharge were assessed on a binary scale, i.e., present or not absent in the sampled cows [49].

Rumen Fill Score is a tool recommended as a key signal for poor health [50,51]. It indicates the total amount of liquid and dry matter in the rumen, and is a function of dry matter intake, feed composition, digestibility, and rate of passage through the gut [52,53,54]. It was visually scored [55], standing behind the cow on the left side and by observing the left paralumbar fossa between the last rib, the lumbar transverse processes, and the hip bone: ‘1’—‘paralumbar fossa empty, presenting a rectangular cavity that is more than a hand’s width behind the last rib and a hand’s width under the lumbar transversal processes’, ‘2’—‘paralumbar fossa forms a triangular cavity with a width about the size of a hand behind the last rib but less than this under the lumbar transverse processes’, ‘3’—‘the paralumbar fossa forms a cavity less than a hand’s width behind the last rib and about a hand’s width vertically downwards from the lumbar transverse processes and then bulges out’, ‘4’—‘the paralumbar fossa skin covers the area behind the last rib and arches immediately outside below the lumbar transverse processes due to a bloated rumen’, ‘5’—‘the rumen is distended and almost fills up the para lumbar fossa, the last rib and the lumbar transverse processes are not visible’.

The consistency of the feces of the sampled cows was visually inspected and rated on a 5-point scale [55]: ‘1’—‘thin and watery and not truly recognizable as feces’, ‘2’—‘thin custard-like consistency, structurally recognizable as feces, splashing out wide upon falling on the floor’, ‘3’—‘thick custard-like consistency, making a plopping sound while falling on the floor and a well-circumscribed pad which spreads out and is about 2 cm thick’, ‘4’—‘stiff with a heavy plopping sound while falling on the floor and a proper circumscribed pad with visible rings and minimal spreading out’, ‘5’—‘hard fecal balls like horse feces’. 

### 2.4. Resource-Based Measures

The total number of sheds per shelter and the number of animals per shed in the shelter was assessed by visual inspection (maximum two sheds per shelter). The length, breadth, and height of the sheds were recorded using a laser distance meter (CP-3007 model, Ultrasonic distance meter 40 KHz frequency, Chullora, New South Wales, Australia) and confirmed using a traditional measuring tape each time. From these measurements, the area of the shed and area per cow was calculated. The space allowance per cow, in shelters with loose housing, was calculated by dividing the floor area of the shed by the total number of cows within the shed. In shelters with stalls, the area per cow was calculated by calculating the floor area of each stall housing a cow [30,56]. In the tethered stalls, the area per cow was calculated by measuring the distance from the end of the rope at the point of attachment, to a peg at the end of the hind limb of the cow, at full extension. This length was used as a radius to calculate the maximum potential area of movement of the tethered cows in the sheds. 

Luminosity in the sheds was measured [57] using a light meter (9V LCD Digital Lux Light Meter Tester LX1010B 0 with 100,000 FC Photo Camera, China), pointed in all six possible directions of the face of a cube, from the center of the shed. The mean of the six readings was calculated for each shelter. Dry bulb temperature and humidity percentage were recorded using a digital meter (TS-FT0423 Digital Wireless Indoor Outdoor Thermo-Hygrometer Thermometer Humidity Meter, Sydney, Australia) inside the shelters, on both days of the study, before any cows were removed. The gradient of the floors in the sheds and the yards were measured at three different places, using vertical and horizontal measurements at each place, using an inclinometer (Bosch Professional, 600MM, DNM60L Model, Australia).

Noise levels [57] were measured at three different locations in the sheds and yards, using an Android phone application [58] (Decibel X). The slipperiness of the floors was determined as the coefficient of friction (CoF) (the force required to move an object over a floor divided by the weight of that object [59,60]). This was estimated using a 1 kg/10 N spring balance attached by a hook to a cuboid wooden block (mass 156 g). The block was gently pulled across the floor, at a speed of 0.17 m/s, and the minimal frictional force (F) required to keep it moving was recorded. 

The number of sides of the sheds that were open, the type of housing (free stall, tie stall, loose, tethered, or no housing) [57], type of roofing (portal, flat, sloped, or other), type of shed flooring (brick, stone, earthen, concrete, or other), presence of bedding in the sheds (present or absent), type of bedding if present (hay, straw, rubber mats, or other), presence of any sharp objects protruding from shed walls or shed furniture, presence of yards and number of trees in the shelter yards [29,30,57,61], watering provisions and the number and types of water points (troughs, bowls, natural water bodies, or other), were recorded in all sheds or yards [56,61]. The appearance of water available to the cows (clear, hazy, or opaque), and the presence of any algal growth [30] were recorded, during the inspection of the shelter facilities.

The cleanliness of the shelter premises was recorded, by visually assessing the mean percentage of the floor that was covered by dung and urine in the sheds, passages, and the yards, separately [62]. Moldiness of each feed offered to the cows in the shelters was assessed by visual inspection and by smelling a sample (recorded as ‘not moldy’ or ‘moldy’). Dustiness (‘not dusty’, ‘dusty’ or ‘very dusty’) of the fodder was assessed by dropping the fodder on the floor from the hand of the assessor. The moisture content of the fodder was assessed on a three-point scale of wet, moist, or dry, through the squeeze test [63], in which the fodder was firmly squeezed in the hand of the assessor and any liquid expression, wetting in the inside of the fist, sticking of the fodder particles to the palm, or presence of a dry palm, was observed. 

## 3. Data Handling and Statistical Analysis

The recordings and observations obtained from the 54 cow shelters (gaushalas) were collated, cleaned for errors, and entered into spreadsheets. Variables were tested for normal distribution by visual inspection and the Anderson–Darling test [64], and data considered to be approximately normally distributed were expressed in terms of a mean value per shelter, standard deviation, and *p*-value for both continuous and categorical data. For data with skewed distributions, the results were expressed as percentages or proportions, as well as median value per shelter. Interquartile ranges (IQR) for the continuous variables and the maximum and minimum values for the categorical variables have been provided. All the analyses were run at a 5% level of significance, for assessment of normality of the distribution of the data, using the statistics software Minitab (Minitab^®^ version 17.1.0, Minitab Ltd, Pennsylvania State University, USA).

## 4. Results

The time required to complete the 40 animal-based measures was approximately 15–20 min per cow, or 8–10 h per shelter. The measurement of resource and management-based parameters took 4 h per shelter. The assessment of each cow shelter, therefore, took 12–14 h. 

### 4.1. Interview with the Shelter Manager

The managers reported a median number of cattle per shelter of 232 (IQR: 587–126) (Table 1). Almost two thirds, 63%, of the cattle in the shelters were cows, the others being bulls, bullocks, calves, and heifers. The median number of cows per shelter was 137 cows (IQR: 272) and the mean age was 11 years. The median mortality incidence rate was 13.6%, with a range of 4% to 76% per year. Only 42% of the cows had identification, in the form of ear tags, and nearly all cows were horned (93.3%). The majority of cows in the shelters were non-lactating (87.9%). Only 26% of the cows examined were classified as aggressive, the remainder being classified as docile. There was a widespread breed distribution, with a predominance of area-specific indigenous Indian breeds including Kankrej, Red Sindhi, Gir, Sahiwal, Dangi, Tharparkar, Deoni, Hariana, Nimari, Khillari, Nagauri, Rathi, Pahari, as well as Holstein Friesian, Jersey, and their cross breeds. The indigenous Indian breed cows comprised 48.6% (787 cows) of the total cows examined, followed by cows that were crossbred with exotic cows 29.1% (472 cows), the cross breeds between indigenous cows 21.5% (349 cows), and the pure-breed exotics 0.7% (12 cows).

The majority of cows (98.2%) had not been screened for tuberculosis and brucellosis. Raw milk was sold in 37% of the shelters to the general public in the open market. Most (92%, *n* = 49) of the gaushalas routinely dewormed the cows, but only 33% had a proper veterinary-prescribed deworming protocol.

Most (72.2%) shelters disposed of cow dung as organic manure to farmers or used it for fertilizing their own pastures; 13% utilized it for biogas production, and 27.7% did not utilize it and just collected it in mounds. Some shelters (20.3%) sold urine as a traditional medicine; most (75.9%) were just allowing the urine to flow out of their premises without proper sewerage disposal facilities. 

Most (96.3%) cows were vaccinated against foot and mouth disease (FMD), hemorrhagic septicemia (HS), and black quarter (BQ), with 79.6% of these being vaccinated biannually. Ectoparasiticidal drugs were administered to 88.8% of cows and endoparaciticidal drugs to 92.5%, on a routine basis; 72.2% of shelters utilized the services of visiting veterinarians in emergencies, while 22.2% had their own veterinarians to treat their cows. 

Carcasses were usually disposed of by burial within the shelter premises (53.7%) or through municipal contractors (40.7%), while a few shelters (5.5%) discarded carcasses into the open. About half (46.3%) of the shelters had biosecurity measures for the introduction of new animals into the shelter and 70.3% had isolation rooms for diseased cows. Some (11.8%) shelters have had disease outbreaks in the last 5 years, primarily FMD.

### 4.2. Animal-Based Measures

The median CCI was 0.27, i.e., a median of 27% of the cows were lying down. Some 31.5% of the cows had an avoidance distance between 50 cm to 0 cm, and 51.2% of the cows allowed touch by the assessor. The BCS of 53.4% of the cows fell in the range of 2–2.75 and the mean BCS on the 1–5 scale was 2.6.

Lameness was rare; only 4.3% of the cows in all the 54 shelters examined had clinical lameness (lameness score >2), while 84.8% of the cows were not lame at all (score 1). The mean score of lameness on the 5-point scale was found to be between 1 and 2 (1.133) (Table 1). More than half (52.47%) of the cows had no claw overgrowth, and 36.3% of the cows had mild claw overgrowth. Severe claw overgrowth (score 3) was observed in just 25 cows (1.5%). 

The rising behavior of cows was mostly normal; 83.6% of the cows rose easily (score 1) and only 10% of the cows had slightly interfered rising behavior (score 2). Similarly, 96.8% of the cows were able to rise without any restriction (score 0), due to the shelter design or presence of furniture.

Medium swellings of the hock joints were detected in 63.7% of cows and almost one half (49.4%) had mild hair loss (<2 cm) in this joint; only 23% of cows had no loss of hair in the hock joints. One-third of the cows (33.3%) had mild levels (<2 cm) of ulcerated hocks, and more than one half (53.6%) had no hock joint ulceration. Carpal joint injuries were also common; only 45% of cows had no evidence of these (score 0) and 55% had hairless and swollen carpal joints (scores 1 and 2). 

The dirtiness of the flanks, udder, and hind limbs of the cows was in the mild to medium range (scores 1 or 2, 74.2%, 76%, and 86% for the three body regions, respectively). The scores for body hair loss of the cows were mostly (53.2% of cows) mild to medium; almost half (45.0%) had no body hair loss. Hair coat condition was almost equally dull and short (47.1% of cows), and shiny and short (52.9%). Ectoparasitism was mostly either absent (53.5%) or mild (34.5%), being mainly lice and ticks in the regions of the tail, croup, udder, groin, and between the elbows and the neck. The skin tenting time was below or equal to two seconds in 92.2% of the cows (score 0). Lesions from the shelter furniture ranged between the absence of lesions (score 0) in 43.8%, mild lesions (score 1) in 37%, and moderate lesions (score 2) in 19% of cows, respectively. 

Neck lesions in the form of hairless patches, swellings, and wounds were found in very few cows (4.5%), most being hairless patches (3.8%; score 1 and 2). Similarly, ocular lesions were observed in only 0.6% of cows, comprising mainly ocular discharges and occasional corneal opacities. There were very few oral lesions (0.05%).

A vast majority of cows (83%) had dry udders and teats (score 1). Chronic udder and teat conditions, like teat and udder fibrosis, and udder abscess, were found in only 1.5% (24 cows) and 0.43% (7 cows) had teat warts. Vulval discharge was observed in 1.6% cows (score 0), predominantly purulent.

The other animal-based health measures, for which we found a low prevalence, were cows with a nasal discharge (9.26%), hampered respiration (0.43%), coughing (proportion of selected cows coughing during the entire cow examination period 0.31%), and diarrhea (4.26%). The Rumen Fill Score revealed a majority of the cows in the score range of 3 (37%) and 4 (59%). The consistency of feces was predominantly in the score range of 3 (35.12%) and 4 (58.27%).

### 4.3. Housing 

The majority of the cow shelters (74%) had one or two sheds for housing the cows, 15% of shelters had between 3 to 9 sheds, and 11% had more than 10 sheds. Most of the cow shelters had none or just one of the sides open (72%), whereas only five shelters (9.2%) had no walls in any of their sheds. There was a predominance of loose (42.5%) or free stall housing (20.3%). Tethered stalls were found in 20 shelters (37%). Almost half of the shelters had concrete flooring (42 out of 86 shelters), almost a quarter had earthen floors (21 out of 86 shelters), followed by brick floors (22%, 19 out of 86 shelters) and stone floors (4%, 4 out of 86 shelters), respectively. Most cow shelters (87%) had yards for cows within their premises, with four different types of materials for the floor (earthen—41 shelters, brick—13 shelters, stone—3 shelters, concrete—19 shelters, out of total 76 shelters).

Portal frames were the most common roofing system (46%), with some flat (29%), sloped (26.7%), and domed (2.3%) roofing systems. Most shelters (54%) used galvanized iron sheets as roofing material, followed by re-enforced concrete cement roofs (32%); a few shelters had thatched roofs made of locally available grasses (7%) or corrugated cement sheets (4.6%). The median height of the roof shed was 3.8 m.

Some sheds (26%) had sharp objects protruding from shed walls or shed furniture. There was no bedding provided in most shelters (97%). Regarding shade provision, most shelters (84%) had none in their yards, and 43% of shelters had no trees in the yards (33% had up to 10 trees). Most shelters (60%) did not provide access to pastures for the cows; 23% provided it for up to 6 h/d, 17% provided access for 7–12 h/d. Free 24-h access to a yard was provided in 30% of the shelters, 29% provided access for up to 6 hours and 27.5% for 7–16h/day; 13.5% of the shelters had no yards at all. 

The median number of sheds per shelter was 2 and the median number of cows per shelter was 70. The median area of shed per cow was 2.73 m^2^ and the yard was 5.9 m^2^. The mean area for tethered cows was 4.50 m^2^. The median luminosity inside the sheds was 582 lux, and the noise levels inside the sheds and yards were 27.7 and 25.3 decibels, respectively. The CoF of the floor passages of the sheds and yards were 0.43 and 0.64, respectively.

### 4.4. Water Provision

Water points in the sheds were absent in 71% of the shelters; if they were present they were predominantly troughs (98%). We observed several different water sources—motorized tube wells (37%) and natural water bodies (ponds, rivers, and wells, 23%). A few shelters had a combination of tubewell and municipal tap water (15%), and 4 shelters offered human-potable water to the cows. Just over one-half of the cow shelters provided ad libitum water (52%), the others mostly (64%) provided it twice a day, 32% provided water three times a day, and one shelter provided water four times a day. One-half of the shelters had water with a hazy appearance, and in the other half, it was clear, none having an opaque appearance. Only 10% had algal growth in the water. Eleven shelters (23%) had no water in the yard and 67% had one or two water points in the yards; nearly all (77%) were troughs. There was a clear appearance of water in the yards for 42% of shelters and only one shelter had opaque water. 

### 4.5. Cleanliness 

A median 20%, 15%, and 10% of the yard, lying area, and passages of sheds, respectively, had dung on the floor (Table 2). In the majority of shelters (83%), no urine was found in the lying areas and the passages of sheds; 11% of the shelter yards had floors with urine. The yards, sheds, and passages were cleaned in 71% of the shelters. Shelter sheds and yards were cleaned once a day in 32% of shelters and twice a day in 39%, usually (87%) by manual floor scraping, but 7% of shelters relied on floor scraping by tractors, and 5.5% used both. 

### 4.6. Feeding

Cows were either fed thrice (54%) or twice daily (45%). The mean quantity of roughage provided was 17.66 kg/cow/d. Most were (78%) fed dry fodder feed and only 17% were fed moist fodder. Moldiness of the fodder was detected in 2% of the shelters, but 27% were fed dusty fodder. A wide range of feeding practices was noticed in the shelters, all relying on wheat, paddy, or millet straw, and these were classified as follows, into four types (with the number of shelters and percentage of shelters):

Dry straw only (*n* = 10, 18.52%)

Dry straw + agricultural by-product waste (*n* = 11, 20.37%)

Dry straw + agricultural by-product waste + hay (*n* = 25, 46.30%)

Dry straw + agricultural by-product waste + hay + greens (tree leaves, vegetables) (*n* = 8, 14.81%)

Concentrate feeding was practiced in 85% of shelters, but in 13% of shelters, there was no processing, by rolling, grinding, or making into pellets. The processing of green and dry roughage involved chopping their stems into smaller pieces, either manually or by a chaff cutter. We categorized the processing practices into 6 types:

No processing (12.96%)

1—Chopping only (14.81%)
2—Chopping + ground concentrate (44.44%)
3—Chopping + cakes (11.11%)
4—Chopping + ground concentrate + cakes (3.70%)
5—Chopping + TMR + Cooked concentrates (7.41%)
6—Chopping + TMR + Cooked concentrates + mineral mixture (5.56%)

## 5. Discussion

### 5.1. Assessment Time

We aimed to assess the conditions of cow shelters (gaushalas) in India. Every effort was made to maintain uniform timing of assessment in all shelters, a potential confounding factor, but a mean temperature difference of only 5 °C was observed between the first and second day of assessment in each shelter. The time duration required to complete the assessment of a shelter was more than that taken by other researchers in their assessments, but the latter generally included only animal-based measurements [25,64,65,66]. Our study involved shelters with a wide variation in herd size, in contrast to other assessments, which had a narrower range of cows per farm [26,32].

### 5.2. Animal-Based Assessment

The mean age of cows was almost 11 years, which is an old age for cattle, compared to the production industries [67], but it demonstrates that the shelters are being used for their intended purpose, to shelter old cows. Mortality is usually an important indicator of poor animal welfare [68,69,70]. The mortality rate in this study (14%) was greater than that of dairy herds in developed countries, even though there has been an increasing trend there [71,72,73]. A mortality rate of 15–20% has been reported in older beef cows (10 years and above), in Australian herds with an overall range of 2% to 12% [74]. However, cows in developed countries are usually sold for slaughter when their productivity declines, or they are diseased. The relatively old age at which abandoned, infirm, and rescued cows enter shelters in India (typically 7–8 years) suggests that mortality is likely to be higher than in dairy farms. Amble and Jain [75] reported a mortality rate of 2% to 6% in cross bred and pure bred cows, in military farms in India, comparable with dairy herds in developed countries [71,72,73,76,77].

Most of the shelter cows were not lactating, so the majority of the cows had dry udders and teats. This parameter has not been assessed in any protocol for dairy cows to date. There are studies on clinical mastitis in Indian cows in peri-urban areas, which report an incidence rate of 1–10%; there is a lower incidence in indigenous cows than in cross breeds and exotics [78]. The reason for the low incidence of mastitis found in our study could be that the vast majority of cows were local low milk yielding breeds.

The general temperament of the cows examined in our study was docile, agreeing with other studies of Indian cattle [79,80], perhaps because of the regular handling, which is normal in India. The human–animal relationship in most shelters was good, as more than half of the cows did not show fear towards the human approach. Additionally, most of the cows were non-lactating, leading to a reduced level of human–animal contact, so the low avoidance scores reflected good stock-personship, despite the cows being of no commercial value. The avoidance distance values found in this study were similar to those of European dairy cattle [45,81]. 

Lying behavior might be one welfare concern in the Indian shelters; the Cow Comfort Index (CCI) was low in comparison to the target of 0.85, which is suggested for dairy cows [82,83]. Reduced lying might be attributed to high stocking density, poor design of the stalls, and the flooring of the sheds. The recommended area per cow is dependent on the size of the animals and the type of shed [84]. In India, the recommended area per cow is 7 m^2^ [85]. In the studied shelters, it was much less, 2.5–6 m^2^ per head. This lower area per cow in the shelters suggests a poor welfare, potentially affecting the behavior and feed access for the cows [86]. The marginally lower than normal BCS in the shelter cows revealed some inadequacies to cow nutrition, which might be due to reliance on low quality straw. 

Lameness has been regarded as one of the most important welfare issues in European dairy cattle, due to economic losses and pain [42,87], and is a key indicator of welfare [45], usually assessed through locomotion scoring [37]. The low incidence of lameness in shelter cows, as compared to lactating dairy cows could be attributed to the feeding of roughage diets to the shelter cows, rather than the high energy diets fed to dairy cows, for milk production. A lameness prevalence rate of 11% has been reported in the French dairy cows [49], and an incidence of 8.1% to 30.5% has been reported in cross bred Indian dairy cows [88,89]. Claw overgrowth was also low, attributable to the low growth rates, and the reasonable floor abrasion [90,91].

The movement and socialization of the cows led to an increased incidence of injuries, disease, and subsequently reduced welfare [92]. Injuries also reflected physical stress from the environment [93]. Joint injuries occurred due to the restrictions of floor space and lying areas [94], and the lack of bedding. In our clinical examinations, joint swellings, hair loss, ulcerations, and injuries of tarsal, hock and carpal joints were at low to moderate levels, probably reflecting the lack of forced movement. These results were in contrast to the studies on the prevalence of hock lesions in the U.K. dairy cows [95]. Soft tissue injuries were also a consequence of improper construction of barns, and aggression between cows in a loose housing system [96,97]. The mild to moderate levels of soft tissue lesions in half of our cows was due to the presence of sharp objects and improper furniture fittings in some of the shelters, as well as aggression between them. The area per cow in the shelters was small, and this overcrowding increased the chances of sustaining injuries. Likewise, competing for fodder at the manger in the limited space, further increased injuries, for example, due to butting by horns, being pushed against shed walls, and sustaining injuries from shelter furniture. Sustaining injuries in a restricted/confined environment, where cows were allowed to interact with each other in a loose housing system, was an inherent problem in the shelters. Overcrowding revealed the shortcomings of flooring, barn fittings, and narrow passages, which were the main potential sources of getting injured. The location of lesions on the body and their contour/shape (lacerations, bruises) was the best indication that they were sustained from shelter furniture and sharp objects. We observed in some shelters that almost all the cows had similar lesions at similar body locations, and we were able to locate their origin, in the form of protruding nails, galvanized sheets, and exposed concrete reinforcement, as well as old mangers protruding from the wall and old gates.

The overall cleanliness levels of the cows in the shelters were much better than that has been observed for dairy cow cleanliness in the U.K. [43] and Eastern Europe [45]. We measured the cleanliness levels of hind limbs, udder, and flanks, as the scoring of these reflected the sources of contamination—dirty legs indicate fecal soiling from waste passage, a dirty tail indicates loose feces, or more time spent in waste passage, and dirty flanks indicate dirtiness of bedding or the tail [98]. Therefore, the cleanliness of our cows reflected that of the shelters, which probably derived from the relatively high labor input into cleaning. The hair coat was also assessed to find out whether the cows were able to maintain their own cleanliness [35]. A lack of self-grooming was indicative of illness, poor general health, and movement restrictions [45]. The dull coat condition of nearly half of the cows (47.1%) of the cows in the shelters reflected their sub-optimal health status. This finding was further strengthened by the marginal BCS found in some shelter cows. 

Dairy cows with tick lesions have been shown to express more kicking behavior and a higher avoidance distance [39]. The prevalence of ectoparasites (46.3%) in the form of ticks, flies, and lice in our survey was lower than that found by Chavhan et al. [99] (77.2–84.8% prevalence in one of the states that we recorded). The negligible presence of neck lesions (4.6%) in our study was probably due to the absence of feed barriers in cow shelters. This is in contrast to the findings in Norwegian dairy cows where neck lesions were observed in 20% to 40% of cows, depending upon the type of feed barriers being used [48]. The proportion of cows showing ocular discharge/lesions, hampered respiration, coughing, and vulvar discharge was higher than in a study on French dairy cows [49]. However, the proportion of cows suffering from diarrhea and showing nasal discharge was less than that in the French study. The incidence of nasal discharge and diarrhea was much less than the threshold limits (to trigger a need for veterinary aid) of Welfare Quality^®^ assessments in Europe. A low frequency of nasal and ocular discharge was also found in the welfare assessment of Danish dairy herds and this was influenced by season [30]. We could not rule out a seasonal influence in the cows we assessed, but were unable to determine that.

### 5.3. Assessment of Disease Status and Carcass Disposal Risks

Regarding the presence of diseases in the cattle, although brucellosis, leptospirosis, and tuberculosis have been reported to be prevalent in cattle in India [100,101,102], most of the cow shelters did not have any testing protocols for the diagnosis of these diseases. Most shelters followed deworming and vaccination practices, routinely, according to the standards laid down by the National Code of Practices for the management of dairy animals in India [103]. Outbreaks of foot and mouth disease (FMD) were the only disease outbreaks, reported by 22 shelters (12%), in the last five years. 

There was no proper provision for disposal of carcasses, dung, and urine, in the majority of the shelters. Carcass disposal by contractors was questionable, as deskinned carcasses were left in the open in some shelters; this is relevant to animal welfare because diseases, such as botulism, could be transferred to other cattle, if they are not disposed off, appropriately, usually by burying. Disease risks associated with improper disposal of urine, feces, and carcasses of livestock, have been emphasized by many workers in Indian conditions [104,105], as they contaminate the groundwater supply, due to the presence of inorganic pollutants and coliform bacteria [106]. 

### 5.4. Housing and Flooring

The five freedoms for good animal welfare must be achieved through the adequate design of housing and other structures, as well as good management practices [107]. Traditionally, there has been a predominance of tethered/tie stalls in Asia [108], but our experience is that these are slowly moving towards loose housing or free stalls, due to the benefits of allowing animals the freedom to move about. Tethered stalls decrease the labor efficiency [60], which is a critical aspect of shelter management in a time when commercial aspects of cow keeping are paramount. The predominance of loose housing in our study indicated a good welfare, as cows were free to move about, but overcrowding might thwart this.

The floor is the primary point of contact of a cow with its environment and is very important for the cow’s movement. It affects wearing of the hooves and conducts heat from the body, when the cow is lying down [60]. Slippery floors affect the behavior and can lead to injuries due to falls [109,110,111]. Earthen flooring is a typical feature of Indian cattle housing. The coefficient of friction values of the yard and shed flooring in our study were higher than those of Telezhenko et al. [112], who reported decreased values in floors made of concrete, asphalt, and rubber, in dairy farms. Appropriate friction levels of the flooring are important to facilitate a comfortable movement of the cows, without slipping, as they provide an adequate grip for the cows’ hooves. Based on the comparisons of the coefficient of friction found in our study, we concluded that the floors were less slippery than in dairy farms [112]. This might be due to lesser movement of the cows, in and out of the sheds, compared with dairy farms, in which the cows are usually moved in and out twice daily. Moreover, access to yards in most shelters reduced the wear of the shed floors. The absence of bedding for cows in the shelters is a significant welfare issue, as it reduces their comfort levels—few cows like to lie down on a non-bedded floor [113]. The body hair loss observed in the cows could be due to the lack of bedding in most of the shelters. The scarcity of fodder straw and its exorbitant cost could be attributed as a factor for the lack of bedding.

The minimum recommended eave height of cattle sheds is 3.5 m [84] and the median height of the sheds in our study (3.8 m) was just above this recommendation, enabling machines to achieve a proper clearance, and work inside sheds. The gradient of lying areas and yards in the shelter sheds was within the recommendations (covered areas 0.5–1.5%; uncovered areas 1–2%), whereas the gradient of passages, which were predominantly in uncovered areas, was similar (1.5%) to the recommendations [84]. A minimum slope of 0.5% (1:200) was recommended, to prevent water pooling, though the floor slope depended on the natural slope of the site and the method of cleaning the floor [108]. A proper gradient was very important for adequate drainage of urine. Most of the shelters in our study had an adequate gradient of the floors, which allowed proper drainage, as the majority of the shelters did not have urine pooling in the lying areas and passages.

### 5.5. Access to Pastures and Yards

Access to pastures is a very important welfare provision for cattle, and deprivation of grazing leads to behavioral and health problems, such as stereotypies, aggression, and lameness [60]. An 8–12 h per day grazing period is considered adequate for cows [60]. In our study, very few shelters had a provision of pasturing for the cows, probably because of lack of resources for this. The yard access provided to the cows in more than half of the shelters would provide some relief to the discomfort experienced in the sheds and reduce the aggressive interactions between the cows. The cow’s heel and heel bulb were weakened by constant hoof contact with the wet flooring, contaminated by the acidic dung where there was no access to pastures or yards. This caused necrosis, digital dermatitis, and laminitis, due to the proteolytic action of the acidic excreta [50]. The comparatively low incidence of lameness and claw overgrowth in the shelter cows testified to the significance of access to the yards and the relative absence of slurry in the lying areas and passages.

### 5.6. Noise and Luminosity Levels

Cows are able to hear higher frequency sounds than humans [114]. This might disturb them and as they lack the capacity to know the direction of the sound as accurately as humans, they might be stressed by being unable to avoid it [60]. The noise levels in shelter sheds and yards recorded in this study were a maximum of 37.7 dB, well below the permissible limits of 90–100 dB [60]. Most shelters in rural areas were located in quiet areas away from the population and the automobile traffic. Cleaning operations were mostly manual, leading to more settled cows than in the commercial dairy sector. 

Light is another important factor regulating animal health and welfare [115]. Light intensity should be between 161 and 215 Lux, during the day [116]. The luminosity levels for the cows in the shelter shed, during the day, were much higher than these levels and stood in contrast to very low levels of light intensity (52–53 Lux) in the studies conducted in Eastern European dairy farms [117]. 

### 5.7. Feeding and Watering Provisions

A dry matter intake of 3% of body weight for dry cows in Indian conditions has been recommended [118], usually achieved by feeding roughages (green and dry) and concentrates (grains, oilcakes, and agricultural by-products) [103]. Birthal [119] in a field survey of dry cows kept in households in rural India, found that the mean daily consumption rates of dry roughage, green roughage, and concentrates were 4.0, 3.4, and 0.4 kg per cow per day, respectively. The dry roughages and greens fed to the gaushala cows in our study appeared to be better than that fed to the dry cows of rural farmers in India. The proportion of cows with a normal rumen fill score in our study, suggests an adequate dry matter intake (DMI), and is comparatively greater than that recorded for dairy cows in England [43]. Fecal consistency indicates the ratio of water intake to dry matter and indirectly provides information about the nutritional and digestive states of cows [55,120]. A score of 3 is an ideal score and indicates a well-digested fodder, a score of 4 is acceptable for dry cows; these were the predominant scores in our sheltered cows. However, the absence of water points inside the sheds, availability of clean drinking water in only 42% of the shelters, and the absence of ad-lib water availability in 48% of the shelters, is a welfare concern. Nevertheless, the majority of the cows assessed in the shelters (92.2%) showed adequate hydration levels, according to the reference scale [47]. It could be due to a better water conservation capacity, which enables the local Indian cattle breeds to withstand dehydration and thermal stress [121].

## 6. Conclusions

Assessing animal welfare using animal-based, resource-based, and management-based assessment tools provided a holistic view of the welfare state of facilities. In this study of welfare assessment of cows in shelters in India, the three types of assessments provided an overview of the welfare conditions and management practices in the shelters, facilitating a diagnosis of conditions for the cows in these shelters. In all shelters, there were several concerns that needed improvement or rectification. These included the small space allowance per cow, non-uniform type of floors, some cows with poor body conditions, little freedom of movement, lack of pasture grazing, lack of bedding, the absence of ad libitum access to water, and compromised biosecurity. The high mortality rate, when compared to commercial dairy farms, is not considered a welfare problem, because many cows enter in poor condition, at an old age.

This study is a scientific assessment of animal welfare and animal management in a specific socio-religious setting. It helped us identify problems directly concerning the cows, which could be used in the future to provide feedback to the shelter managers, for rectification and improvement of their institutions. The purpose of the shelters is to house unwanted cows to the highest standards of animal welfare, despite their commercial redundancy. This is in keeping with the tradition and religious sentiments of India, which espouses the holiness of the cows. The results of this study revealed varying levels of welfare of cows in Indian shelters, which partly contradicts our original hypothesis that these unproductive, old, infirm, and abandoned cows would suffer from poor welfare practices and conditions. Continuous efforts are required by stakeholders to develop new, sustainable management practices, and optimize the existing ones, to improve the welfare outcomes in the shelter cows. Further research is needed to investigate the interplay of the various welfare parameters and to identify their association with the risk factors we identified. We recommend an ongoing work on the repeatability and validity of the assessments. The results of this study can be dovetailed into a restructuring of the gaushalas on scientific lines, based on global animal welfare practices, to ensure the sustainability of these unique institutions.

## Figures and Tables

**Table 1:** Descriptive statistics for animal-based measures in the cow shelters, measured on ordinal and continuous scales.

Parameter	Mean/Median *	Standard Deviation	First Quartile Q_1_	Third Quartile Q_3_	Interquartile Range IQR *	*p*-Value of Distribution (for Normal Distributed Data)
Total no. cattle in the shelter	232 *	-	126	587	460	
Cows as % of cattle	63.42 *		52.65	73.48	20.84	
No. cows	137 *		77	349	272	
Cow age (years)	11.0	2.02				0. 36
Annual Mortality (%) **	1.14 (13.80)	0.399				0.57
Proportion of cows with identification	0.41 *		0.0	0.82	0.82	
Proportion of horned cows	0.93 *		0.7	1.000	0.3	
Proportion of lactating cows	0.03 *		0.000	0.2	0.2	
Temperament score**	0.41 (2.61)	0.068				0.24
Cow comfort Index (CCI), (no. cows lying/total no. cows)	0.27		0.13	0.34	0.20	
Avoidance Distance (AD) Score (scale 1–4)	1.53 *		1.2	2.13	0.93	
Body Condition Score (BCS) Score (scale 1–5)	2.69	0.366				0.27
Lameness score (scale 1–5)	1.13 *		1.05	1.27	0.22	
Claw overgrowth score (scale 0–3)	0.61 *		0.23	0.90	0.67	
Hock joint swelling score (scale 0–3)	1.64 *		0.233	2.233	0.44	
Hock joint hair loss score (scale 0–3)	1.05	0.298				0.22
Hock joint ulceration score (scale 0–3)	0.59	0.386				0.16
Carpal joint injuries score (scale 0–3)	0.78	0.455				0.17
Dirty hind limbs score ** (scale 0–3)	0.21 ** (1.59)	0.110				0.63
Dirty udder score (scale 0–3)	1.27	0.560				0.90
Dirty flanks score (scale 0–3)	1.24	0.570				0.95
Body hair loss score (scale 0–3)	0.76 *		0.066	2.033	1.04	
Coat condition score (scale 1–3)	1.54	0.298				0.07
Ectoparasitism score (scale 0–3)	1.51 *		0.966	3.267		
Skin tenting score (scale 0–4)	0.03 *		0.000	0.833		
Lesions from shelter furniture score (scale 0–3)	0.75 *		0.066	1.600	0.67	
Teat condition score (scale 0–5)	1.0 *		0.92	1.00	0.075	
Neck lesions score (scale 1–5)	1.03 *		1.000	1.10	0.1	
Ocular lesions score (scale 0–1)	0.06 *		0.033	0.133	0.1	
Nasal discharge score (scale 0–1)	0.05 *		0.000	0.141	0.141	
Rumen Fill Score (scale 1–5)	3.7 *		3.19	3.90	0.708	
Faecal consistency score (scale 0–5)	3.70 *		3.19	3.93	0.741	
Diarrhoea score (scale 0–1)	0.000 *		0.000	0.033	0.033	

* non-normally distributed data, ** Log_10_ transform.

**Table 2:** Median, first quartile (Q_1_), third quartile (Q_3_), and interquartile range (IQR) values for the non-normally distributed data, and mean, standard deviation (SD), and *p*-values for the normally distributed data, for resource-based parameters of cows in shelters.

Variable	Median/Mean *	SD	First Quartile Q_1_	Third Quartile Q_3_	Inter Quartile Range IQR	*p*-Value (Normal Distribution)
Total number of sheds	2.0		2	4	2	
Number of animals/shed	70.0		48.8	137.3	88.5	
Area of the shed (m^2^)	173		99	313	214	
Area of the yard (m^2^)	756		178	1800	1622	
Shed Area/cow (m^2^/cow)	2.73		1.56	3.63	2.07	
Yard Area/cow (m^2^/cow)	5.9		3.6	21.5	17.9	
Area of movement of tethered cows (m^2^)	4.50 *	2.752				0.044
Height of eaves in sheds (m)	3.80		2.99	5.34	2.35	
Luminosity in sheds (Lux)	582		89	1036	946	
Noise levels in sheds (Decibels)	27.67		21.33	37.17	15.83	
Noise levels in the yards (Decibels)	25.33		20.33	33.00	12.67	
Dry bulb reading in sheds (◦C)	29.50		27.2	32.8	5.6	
Humidity in sheds (%)	34.00		24.7	45.2	20.5	
Coefficient of friction in shed passage floors	0.43		0.27	0.65	0.37	
Coefficient of friction in yard passage floors	0.64		0.34	0.68	0.34	
Mean gradient of shed lying areas	1.46		0.96	2.2	1.23	
Mean gradient of shed passages	2.36		1.27	3.52	2.24	
Mean gradient of the yard floors	1.51		1.13	2.43	1.30	
Percent dung in lying areas of sheds	15.00		5.00	40.00	35.00	
Percent dung in the passages of sheds	10.00		5.00	42.50	37.50	
Percent dung in yards	20.00		10.00	40.00	30.00	
Quantity of roughages provided to the cows (kg) **	1.25 ** (17.66)	0.168				0.061

* Mean; ** Log transformed.

## Appendix A

**Table A1:** Animal-based parameters used for the assessment of the welfare of cows in Indian shelters.

Parameter	Description	Scales and Scores
General temperament [31]	Visual examination	0- docile 1- aggressive
Cow Comfort Index (CCI) [32]	Proportion of cows in a stall or shed that were lying down	
Avoidance Distance (AD) [33]	Cows that were standing at the feeding manger were approached at the front at a rate of one step per second, starting at 2 m from the manger. The distance between the assessor’s hand and the cow’s head was estimated at the moment the cow moved away and turned its head	0- touched 1- 0 to 50 cm 2- 51 to 100 cm 3- >100 cm
Lactation		0- Non-lactating 1- Lactating
Body Condition Score (BCS) [34,35]	A cow with a score of ≤ 1.25 was considered emaciated, 1.5–2 thin, 2.25–3.75 normal and 4 or more obese Visual examination	1 to 5 with increments of 0.25.
Lameness Score [36]	1 to 5 scale Visual examination	1- not lame (smooth and fluid movement) 2- mildly lame but not observable easily (an imperfect gait but able to freely move with a mildly arched back) 3- moderately lame (able to move but not freely, with an arched back) 4- lame, with inability to move freely with and asymmetrical gait and abnormal head movement 5- severely lame (severely restricted in movement, requiring considerable encouragement to move, and a severely arched back)
Claw overgrowth [37]	Visual examination	0- Normal claws 1- Mild claw overgrowth 2- Moderate claw overgrowth 3- Severe claw overgrowth.
Rising behavior [38,39]	All cows lying in the shelter were coaxed to get up with use of a minimum amount of force. If the presence of the assessor did not evoke rising they were given one or two gentle slaps on the back, followed by a break of 5 s, then more slaps with slightly more force if required, up to a maximum of 30 s	1- Normal (smooth and a normal sequence of rising behaviour 2- Easy but slightly interfered (smooth movement with slight twisting of the head but with normal sequence of rising process 3- Uneasy with effort (sudden movement and difficulty in rising with awkward twisting of the head and neck but following a normal sequential rising process 4- Abnormal (uncharacteristic sequence of a rising event) 5- refused to get up
Rising restrictions [40]	As a result of shelter facilities by visual inspection	0- Unrestricted (cow is able to rise as if it were in a pasture) 1- Mild restrictions (cow is able to modify standing to rise comfortably as it lunges sideways and not forwards) 2- Cow takes time to rise and hits shed fixtures or fittings while rising 3- Dog sitting posture adopted while standing or make multiple attempts before able to rise.
Hock joint swellings [41,42]	Visual examination	1- mild swollen joint 2- medium swollen joint 3- severely swollen joint
Hock joint hair loss and ulceration [41,42]	Visual examination	0- no hair loss or ulceration 1- mild hair loss or ulceration <2 cm^2^ 2- medium hair loss or ulceration (approx. 2.5 cm^2^) 3- severe hair loss or ulceration >2.5 cm^2^
Carpal joint injuries [41]	Visual examination	0- no skin change 1- hairless 2- swollen 3- wound(s)
Dirtiness of the hind limbs, udder and flanks [43]	By visual inspection of the cows from both sides (left and right) and from behind	1- no dirtiness 2- mildly dirty (small soiled areas of dirtiness with no thick scabs) 3- medium dirtiness (large soiled areas but with < 1 cm thick scabs of dung) 4- severely dirty (large soiled areas with > 1cm thick dung scabs)
Body Coat condition [44]	Visual examination	1- dull and short 2- shiny and short 3- dull and hairy
Ectoparasitism [45]	Visual examination	1- Absence of ectoparasites 2- Mild infestation—no lesions (not easily visible by naked eye but on tactile perception in the neck region 3- Moderate-mild infestation visually observable ectoparasites or immature forms or eggs in the neck, groin, peri rectal, tail root and switch regions 4- Severe-Visually observation of mature ectoparasites all over the body especially regions mentioned in score 3
Lesions from shelter furniture [37]	Visual examination	0- normal (no lesions present) 1- small area of hair loss 2- moderate area of hair loss and/or thickening of the skin 3- severe (a large area of hair loss and/or breakage of the skin
Skin lesions/Integument alterations [46]	Visual examination	0- normal (no apparent lesions) 1- mild hair loss (< 2 cm^2^) 2- moderate (> 2 cm^2^ hair loss and inflamed skin) 3- severe (a large > 4 cm^2^ area of hair loss with extensive skin inflammation and breakage)
Teat and udder condition	Visual inspection	1- Normal teats and udder 2- Dry udder and teats 3- Teat cracks 4- Warts on teats and udder 5- Acute lesions on the teats and udder 6- Chronic lesions on teats and udder
Skin tenting time [47]	Visual examination by skin pinch of the cervical region of neck	1- ≤ 2 s 2- >2 s 3- ≥6 s
Oral lesions	Visual examination	0- absent 1- present
Neck lesions [48]	Visual examination	1- no observable skin change 2- hair loss 3- swollen 4- closed wounds (hematomas or closed abscesses) 5- open wounds
Ocular lesions [48]	Visual examination	0- absent 1- present
Nasal discharge [48]	Visual examination	0- absent 1- present
Hampered respiration [48]	Visual examination	0- absent 1- present
Vulvar discharge [48]	Visual examination	0- absent 1- present
Rumen Fill Score [55]	Visually by standing behind the cow on the left side and observing the left para lumbar fossa between the last rib, the lumbar transverse processes and the hip bone	1- the para lumbar fossa is empty, presenting a rectangular cavity that is more than a hand’s width behind the last rib and a hand’s width under the lumbar transversal processes 2- the para lumbar fossa forms a triangular cavity with a width about the size of a hand behind the last rib, but less than this under the lumbar transverse processes 3- the para lumbar fossa forms a cavity less than a hand’s width behind the last rib and about a hand’s width vertically downwards from the lumbar transverse processes and then bulges out 4- the para lumbar fossa skin covers the area behind the last rib and arches immediately outside below the lumbar transverse processes due to a bloated rumen 5- the rumen is distended and almost fills up the para lumbar fossa; the last rib and the lumbar transverse processes are not visible.
Fecal consistency [55]	Visual inspection	1- thin and watery and not truly recognizable as feces 2- thin custard-like consistency, structurally recognizable as feces, splashing out wide upon falling on the floor 3- thick custard-like consistency, making a plopping sound while falling on the floor and a well-circumscribed pad which spreads out and is about 2 cm thick 4- stiff with a heavy plopping sound while falling on the floor and a proper circumscribed pad with visible rings and minimal spreading out 5- hard fecal balls like horse feces
